# Prognostic significance and therapeutic potential of the activation of anaplastic lymphoma kinase/protein kinase B/mammalian target of rapamycin signaling pathway in anaplastic large cell lymphoma

**DOI:** 10.1186/1471-2407-13-471

**Published:** 2013-10-10

**Authors:** Ju Gao, Minzhi Yin, Yiping Zhu, Ling Gu, Yanle Zhang, Qiang Li, Cangsong Jia, Zhigui Ma

**Affiliations:** 1 Department of Pediatrics, West China Second University Hospital, Sichuan University, Section 3, 20 S. Renmin Road, Chengdu 610041, China; 2 Department of Pathology, Shanghai Children’s Medical Center, Shanghai Jiaotong University, Shanghai, China

**Keywords:** Anaplastic large cell lymphoma (ALCL), Anaplastic lymphoma kinase (ALK), AKT, mTOR, 4E-BP1, p-70S6K1, Prognosis

## Abstract

**Backgroud:**

Activation of the protein kinase B/mammalian target of rapamycin (AKT/mTOR) pathway has been demonstrated to be involved in nucleophosmin-anaplastic lymphoma kinase (NPM-ALK)-mediated tumorigenesis in anaplastic large cell lymphoma (ALCL) and correlated with unfavorable outcome in certain types of other cancers. However, the prognostic value of AKT/mTOR activation in ALCL remains to be fully elucidated. In the present study, we aim to address this question from a clinical perspective by comparing the expressions of the AKT/mTOR signaling molecules in ALCL patients and exploring the therapeutic significance of targeting the AKT/mTOR pathway in ALCL.

**Methods:**

A cohort of 103 patients with ALCL was enrolled in the study. Expression of ALK fusion proteins and the AKT/mTOR signaling phosphoproteins was studied by immunohistochemical (IHC) staining. The pathogenic role of ALK fusion proteins and the therapeutic significance of targeting the ATK/mTOR signaling pathway were further investigated in vitro study with an ALK + ALCL cell line and the NPM-ALK transformed BaF3 cells.

**Results:**

ALK expression was detected in 60% of ALCLs, of which 79% exhibited the presence of NPM-ALK, whereas the remaining 21% expressed variant-ALK fusions. Phosphorylation of AKT, mTOR, 4E-binding protein-1 (4E-BP1), and 70 kDa ribosomal protein S6 kinase polypeptide 1 (p70S6K1) was detected in 76%, 80%, 91%, and 93% of ALCL patients, respectively. Both phospho-AKT (p-AKT) and p-mTOR were correlated to ALK expression, and p-mTOR was closely correlated to p-AKT. Both p-4E-BP1 and p-p70S6K1 were correlated to p-mTOR, but were not correlated to the expression of ALK and p-AKT. Clinically, ALK + ALCL occurred more commonly in younger patients, and ALK + ALCL patients had a much better prognosis than ALK-ALCL cases. However, expression of p-AKT, p-mTOR, p-4E-BP1, or p-p70S6K1 did not have an impact on the clinical outcome. Overexpression of NPM-ALK in a nonmalignant murine pro-B lymphoid cell line, BaF3, induced the cells to become cytokine-independent and resistant to glucocorticoids (GCs). Targeting AKT/mTOR inhibited growth and triggered the apoptotic cell death of ALK + ALCL cells and NPM-ALK transformed BaF3 cells, and also reversed GC resistance induced by overexpression of NPM-ALK.

**Conclusions:**

Overexpression of ALK due to chromosomal translocations is seen in the majority of ALCL patients and endows them with a much better prognosis. The AKT/mTOR signaling pathway is highly activated in ALK + ALCL patients and targeting the AKT/mTOR signaling pathway might confer a great therapeutic potential in ALCL.

## Background

Anaplastic large cell lymphoma (ALCL) is an aggressive form of non-Hodgkin’s lymphoma (NHL) of T/null lineage. It constitutes approximately 5% of all human NHL, but accounts for as many as 30% to 40% of pediatric large cell lymphomas [1]. Roughly 50 to 70% of ALCL patients carry characteristic chromosomal translocations involving the anaplastic lymphoma kinase (ALK) gene on chromosome 2p23, resulting in ALK overexpression due to the abnormal fusion of ALK with other genes [2]. The most common of these aberrations is the t(2;5)(p23;q35), which leads to the expression of the chimeric protein nucleophosmin-ALK (NPM-ALK). The others express variant fusions that, like NPM-ALK, involve the entire cytoplasmic portion of ALK, but fuse to other N-terminal partners, like tropomyosin 3 (TPM3) or 5-aminoimidazole-4-carboxamide ribonucleotide formyltransferase/IMP cyclohydrolase (ATIC) [3-5]. So far, at least 15 variant-ALK fusion genes have been identified in both hematopoietic malignancies, such as ALCL and diffuse large B cell lymphoma, and non-hematopoietic neoplasms, including inflammatory myofibroblastic tumor, esophagus cancer, and non-small cell lung cancer [6-11]. ALK immunostainning of NPM-ALK-positive ALCL cases shows a characteristic cytoplasmic and nuclear distribution of the chimeric ALK protein that is due to hetero-oligomerization of NPM-ALK and normal NPM, a phosphoprotein that normally shuttles ribonucleoproteins between the cytoplasm and nucleus, but can aberrantly transport NPM-ALK to the nucleus [12]; whereas patients with the variant-ALK fusion proteins demonstrate cytoplasmic staining only [13,14]. In ALCL, ALK expression has a strong clinical prognostic significance. Compared with ALK negative (ALK-) ALCL, ALK positive (ALK+) ALCL occurs more frequently in younger patients who respond well to chemotherapy and have a favorable clinical outcome [1,15-19].

The mechanisms of cell transformation mediated by the ALK oncoprotein are only partially understood [5,20,21]. However, the NPM-ALK-induced activation of mammalian target of rapamycin (mTOR), either transduced through the mitogen-induced extracellular kinase (MEK)/extracellular signal-regulated kinase (ERK) signaling pathway, or to a much less degree, through the phosphatidylinositol 3-kinase/protein kinase B (PI3K/AKT) pathway has been shown to contribute to the tumorigenesis of ALCL [22,23]. In ALK + ALCL cell lines and tumors, the mTOR signaling proteins, including mTOR, eukaryotic initiation factor 4E-binding protein-1 (4E-BP1), and the 70 kDa ribosomal protein S6 kinase polypeptide 1 (p70S6K1) kinase are highly phosphorylated [23]. Inhibition of mTOR with rapamycin or silencing mTOR gene product expression using mTOR-specific small interfering RNAs decreased phosphorylation of the mTOR signaling proteins and induced cell cycle arrest and apoptosis in ALK + ALCL cells, indicating that blockage of the mTOR signaling pathway represents a potential therapeutic strategy in ALK + ALCL [22,23]. Nevertheless, there is little evidence demonstrating the clinical prognostic value of the mTOR pathway activation in ALCL. In this relatively large case study, we showed that the AKT/mTOR pathway was highly activated in ALK + ALCLs compared with ALK- cases. Phosphorylation of AKT and mTOR was correlated to the expression of ALK, suggesting an activated ALK/AKT/mTOR pathway in ALK + ALCL; and this activation pathway was further confirmed by overexpression of NPM-ALK in the nonmalignant murine pro-B lymphoid cell line, BaF3. In contrast to ALK expression, expression of p-AKT, p-mTOR, p-4E-BP1, or p-p70S6K1 did not have any prognostic significance in ALCL; however, in vitro studies indicated that targeting the hyperactivated AKT/mTOR pathway effectively inhibited cell growth, triggered apoptosis, and reversed glucocorticoid (GC) resistance, suggesting an attractive therapeutic potential of AKT/mTOR inhibitors in ALCL.

## Methods

### Tumor samples

The tumor specimens were obtained from 103 patients with ALCL who underwent biopsy between January 2005 and October 2012 at the Department of Pathology, West China Hospital of Sichuan University, or the Department of Pathology, Shanghai Children’s Medical Center of Shanghai Jiaotong University. None of the patients had received any treatment before surgery. Tumor diagnosis was based on histological examination of tissue specimens obtained by biopsy and based on the criteria of the World Health Organization Classification. Written informed consent was obtained prior to sample collection from all patients or their parents if the patients were young children. This study was approved by the Institutional Review Board of the Ethical Committee of Sichuan University.

### Immunohistochemical (IHC) studies

Rabbit polyclonal antibodies specific for Thr308p-AKT (p-AKT), Ser2448p-mTOR (p-mTOR), Thr70p-4E-BP1 (p-4E-BP1), and Thr421p-p70S6K1 (p-p70S6K1) (Cell Signaling Technology, Beverly, MA) were used. ALK expression was assessed initially by using rabbit polycloncal antibody ALK11 (a kind gift from Dr. Stephan W. Morris,St. Jude Children’s Research Hospital) and further confirmed by the mouse monoclonal antibody ALK-1 (Dako Cytomation, Carpinteria, CA) to exclude false positivity. IHC staining was performed to assess protein expression in formalin-fixed, paraffin embedded samples by the 2-step Envision procedure using a DAKO Autostainer (Dakopatts, Copenhagen, Denmark). The sections (6 μm) were de-paraffinized in xylene, dehydrated through a graded series of alcohol, and immersed for 15 min in phosphate-buffered saline (PBS). For antigen retrieval, sections were boiled in a pressure cooker for 4 min in 0.01 M citrate buffer (pH 6.0). Endogenous peroxidase activity was blocked with 3% hydrogen peroxidase in methanol (10 min), and non-specific staining was then blocked with a 20 min incubation with normal horse serum. The sections were subsequently incubated overnight at 4°C with primary antibodies (ALK11, ×1000; ALK1, ×100; p-AKT, ×100; p-mTOR, ×100; p-4E-BP1, ×50; p-p70S6K1, ×25) in a humid chamber, treated for 30 min with a biotinylated horse secondary antibody against mouse immunoglobulins (ABC Elite; Vector, Burlingame, CA), and then exposed for 5 min to 0.06% diaminobenzidine with 0.01% hydrogen peroxidase. The sections were lightly counterstained with hematoxylin. Controls were performed by omitting the primary antibodies.

Evaluation of the IHC staining was performed in a blinded set up regarding the clinical data. Scoring of the expression was performed semiquantitatively. In brief, both percentage of stained cells and staining intensity were evaluated. No staining or weak staining in <10% of cells was defined as 0, weak staining in at least 10% as 1, moderate staining in up to 50% as 2 and moderate staining in >50% of cells and strong staining of any percentage of the cells as 3.

### Overexpression of *NPM-ALK* in BaF3 cells and targeting of the AKT/mTOR pathway by kinase inhibitors

The murine pro-B cell, BaF3, and an ALK + ALCL cell line, Karpas 299, were kindly provided by Dr. Stephan W. Morris (St. Jude Children’s Research Hospital, Memphis, TN, USA). BaF3 cells (8 × 10^6^) were electroporated with pcDNA3-*NPM-ALK* or empty vector (80 μg DNA, 975 μF, 270 V), then selected in IL-3-containing media with 1 mg/mL G418. G418-resistant pools were tested for NPM-ALK expression, and then seeded at 2 × 10^5^ cells/mL in growth media with or without IL-3. BaF3/NPM-ALK and Karpas 299 cells were maintained in RPMI 1640 (Gibco. Carlsbad, CA, USA) supplemented with 10% fetal bovine serum (FBS; Sigma, St. Louis, MO, USA), 2 mM L-glutamine (Gibco), and antibiotics (penicillin 100 U/ml and streptomycin 50 μg/ml) at 37°C in a humidified 5% CO2 in-air atmosphere. BaF3 cells were cultured in the same media but with 10 ng/ml IL-3.

NVP-BEZ235 was provided by Novartis Pharma AG (Basel, Switzerland). For in-vitro use, NVP-BEZ235 was dissolved in DMSO (Sigma-Aldrich Corp., St. Louis, MO) to a stock concentration of 100 mmol/L, stored at −20°C, and further diluted to an appropriate final concentration in RPMI 1640 at the time of use. Dexamethasone ( Dex, Sigma, St Louis, MO, USA) was dissolved in ethanol and used at the concentration of 1 μM. Logarithmically growing cells were harvested and replated in 96- or 6-well sterile plastic culture plates (Corning) to which 1 mmol/L NVP-BEZ235 (NVP group), 1 μM dexamethasone (Dex group), 1 mmol/L NVP-BEZ235 plus 1 μM dexamethasone (NVP + Dex group), or 0.05% DMSO plus 0.1% ethanol (Con group) was added. At the end of the incubation, cells were transferred to sterile centrifuge tubes, pelleted by centrifugation at 400 g at room temperature for 5 min, and prepared for analysis as described below.

The MTT assay, used to determine the anti-proliferative effect of NVP-BEZ235 on cells growing in culture, together with the apoptosis assay and Western blotting analysis were performed as described previously (24).

### Statistical analysis

Statistical analysis was carried out by using the SPSS 15.0 software package. Correlations between various parameters were calculated by Student’s t-test. Comparison of the expressions of the mTOR signaling proteins between ALK + and ALK- ALCL tumors was performed by chi- squared (χ2) test. The multivariate analysis was performed by the Cox proportional hazards model to identify subsets of independent prognostic factors for overall survival (OS). OS curves were estimated by using the Kaplan–Meier method and the log–rank test was used for comparing survival curves of the two groups. A p value < 0.05 was considered statistically significant. The analysis included mean values, standard deviation, standard error, and a 95% confidence interval.

## Results

### Histology and immunophenotyping of ALCL tumor samples

**Figure 1:**
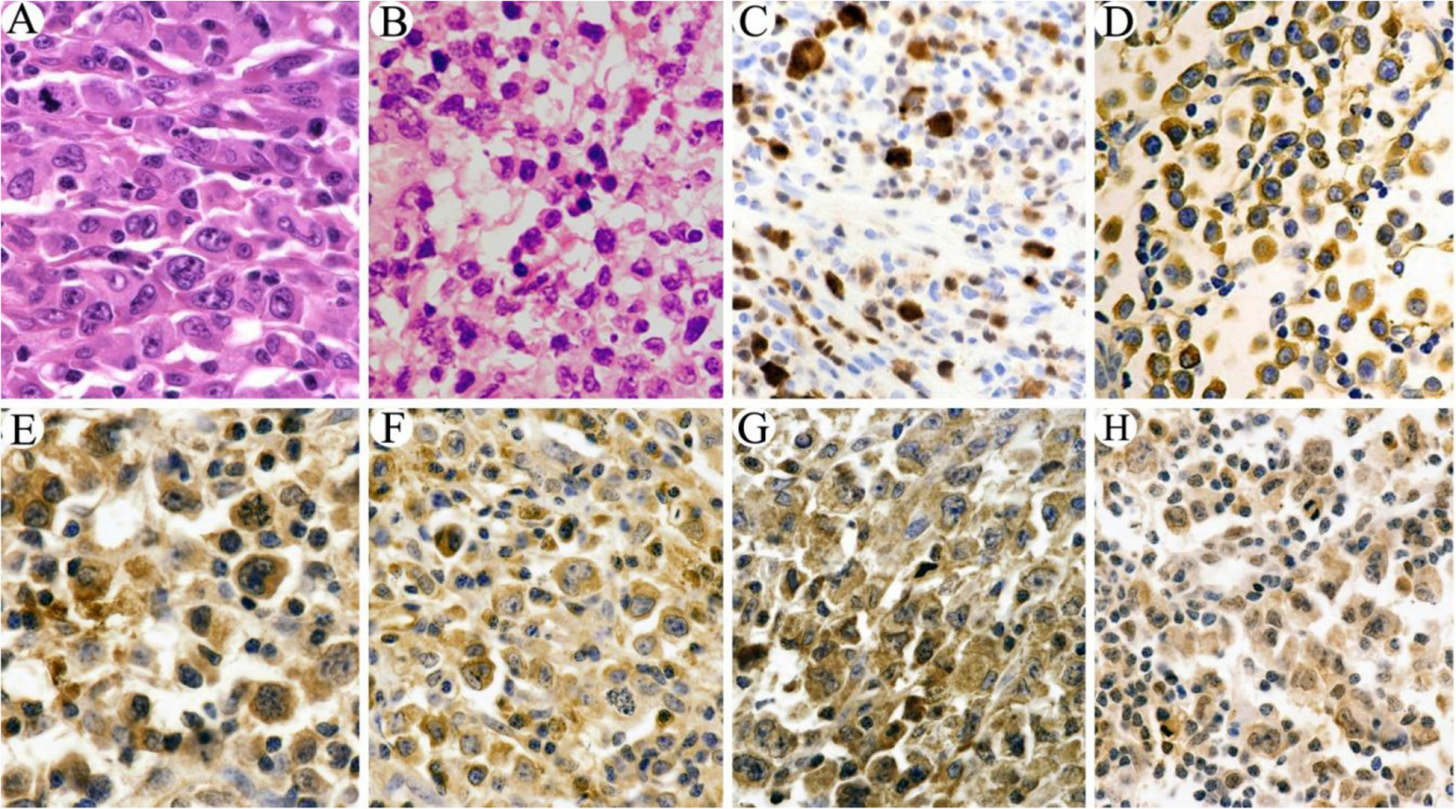
**Histology and immunohistochemistry (IHC) of ALCL tumor samples. (A, B)** Histology of ALCL tumor samples (HE staining). **(A)** The classic type of ALCL. The tumor cells are large with abundant cytoplasm and manifest prominent nucleoli with an eccentrically located and pleomorphic nucleus of kidney-shape. **(B)** ALCL of small cell type. The tumor cells are small in size, with abundant cytoplasm and prominent nucleoli. **(C to H)** IHC staining of ALCL tumor samples (EnVision staining). Expression of ALK, p-AKT, p-mTOR, p-4E-BP1, and p-p70S6K eIF4E was assessed. **(C, D)** IHC staining with ALK1 antibody. **(C)** The tumor cells show cytoplasmic and nuclear staining. **(D)** The tumor cells manifest cytoplasmic staining only. **(E)** IHC staining with Thr308p-AKT antibody. The tumor cells show cytoplasmic and nuclear staining. **(F)** IHC staining with Ser2448p-mTOR antibody. The tumor cells show cytoplasmic staining. **(G)** IHC staining with Thr70p-4E-BP1 antibody. The tumor cells show cytoplasmic staining. **(H)** IHC staining with Thr421p-p70S6K antibody. The tumor cells show cytoplasmic staining. All images were captured at 400× magnification.

One hundred and three ALCL tumor samples were histologically divided into 3 types. Eighty cases (78%) were of common or classic type (Figure 1A), twenty (19%) were of small cell type (Figure 1B), and the remaining 2 (2%) cases were of lymphohistiocytic type. All tumor cells strongly expressed CD30. Seventy (68%) were of T-cell type expressing CD3 and/ or CD45RO and the other 33 were of null cell type expressing no T- or B- lineage markers. Eighty two (80%) expressed TIA1/Granzyme B and 62 (60%) cases expressed epithelial membrane antigen (EMA).

### Expression of ALK fusion proteins

In the 103 cases of ALCL, 62 (60%) were ALK positive, and either showed both cytoplasmic and nuclear staining indicative of the presence of NPM-ALK [49 cases, 79%, (Figure 1C), or cytoplasmic staining only indicating variant-ALK fusions 13 cases, 21%, (Figure 1D). Of the ALK + ALCL cases, the percentages of classic, histolymphocytic, and small cell type were 74%, 3%, and 23%, respectively. In the 41 ALK- ALCL patients, the percentages were 83%, 2%, and 15% respectively. There were no statistically significant differences of ALK expression between different histological subtypes of ALCL (χ2 = 0.642, p > 0.05).

### Expression of p-AKT and its correlation to ALK expression

Immunostaining was performed with anti-p-AKT in 71 out of 103 cases of ALCL, 54 (76%) were positive (Figure 1E). In the 54 p-AKT positive (p-AKT+) cases, 38 (70%) were ALK + and 16 (27%) were ALK-. In the 17 p-AKT negative (p-AKT-) cases, 6 (35%) were ALK + and 11 (65%) were ALK-. Expression of p-AKT was correlated to ALK expression (×2 = 6.750, p < 0.05). In the 44 ALK + cases studied with p-AKT staining, 39 (87%) showed activation of AKT. In the 33 ALK + cases with both cytoplasmic and nuclear staining, 29 (88%) were p-AKT + and 4 (12%) were p-AKT-, and in the 11 cases with cytoplasmic staining only, 10 (91%) were p-AKT + and 1 (9%) was p-AKT-. The expression of p-AKT had no correlation to the different ALK expression (p > 0.05; Table 1).

**Table 1:** Relationship between p-AKT expression and ALK activation in ALCL

	**p-AKT + (n = 54)**		**p-AKT- (n = 17)**		**p-value**
	**No. of patients**	**%**	**No. of patients**	**%**	
ALCL (n = 71)					
ALK+	38 of 54	70	6 of 17	35	
ALK-	16 of 54	30	11 of 17	65	0.009*
ALK + (n = 44)					
ALK (n + c)	29 of 39	74	4 of 5	80	
ALK (c)	10 of 39	26	1 of 5	20	0.779

* Statistically significant value.

ALK(n + c) indicates ALK + with both nucleus and cytoplasmic staining; ALK(c), ALK + with cytoplasmic staining only.

### Expression of p-mTOR and its correlation to the expression of ALK and p-AKT

Immunostaining was performed with anti-p-mTOR in 71 cases, 57 (80%) were p-mTOR positive p-mTOR+, (Figure 1F), of which 39 (68%) were ALK+, and 18 (32%) were ALK-; 47 (82%) were p-AKT+, and 10 (18%) were p-AKT-. In the 14 p-mTOR negative (p-mTOR-) cases, 5 (36%) were ALK+, 9 (64%) were ALK-, 7(50%) were p-AKT+, and 7 (50%) were p-AKT-. Expression of p-mTOR was correlated to the expression of both ALK and p-AKT (×2 = 5.102 and 6.501 respectively, p < 0.05). In the 39 ALK + ALCL cases showing cytoplasmic and nuclear staining, 30 (91%) were p-mTOR+, and 3 (9%) were p-mTOR-, and in the 11 ALK + cases showing cytoplasmic staining only, 9 (82%) were p-mTOR + and 2 (18%) were p-mTOR-. The subcellular ALK expression patterns had no impact on the expression of p-mTOR in ALCL (χ2 = 0.619, p > 0.05; Table 2).

**Table 2:** Relationship between p-mTOR expression and activation of ALK and AKT

	**p-mTOR + (n = 57)**		**p-mTOR- (n = 14)**		**p-value**
	**No. of patients**	**%**	**No. of patients**	**%**	
ALK (n = 71)					
ALK+	39 of 57	68	5 of 14	36	0.024*
ALK-	18 of 57	32	9 of 14	64	
p-AKT (n = 71)					
p-AKT+	47 of 57	83	7 of 14	50	0.011*
p-AKT-	10 of 57	17	7 of 14	50	
ALK + (n = 44)					
ALK(n + c)	30 of 39	77	3 of 5	60	0.431
ALK(c)	9 of 39	23	2 of 5	40	

*Statistically significant value.

### Expression of p-4E-BP1 and p-p70S6K1

Sixty-four out of 71 (90%) ALCL tumors studied were p-4E-BP1 positive (p-4E-BP1+, Figure 1G), of which 40 (63%) were ALK+, 50 (78%) were p-AKT+, and 56 (88%) were p-mTOR+. In the 7 p-4E-BP1 negative (p-4E-BP1-) cases, 4 (57%) were ALK+, 4 (57%) were p-AKT+, and 1 (14%) was p-mTOR+. The expression of p-4E-BP1 had no correlation to that of ALK and p-AKT (χ2 = 0.783 and 1.359, respectively, p > 0.05), but was closely related to the expression of p-mTOR (χ2 = 16.531, p < 0.01).

Sixty-six of the 71 (93%) ALCL tumors were p-p70S6K1 positive (p-p70S6K1+, Figure 1H), of which 42 (64%) were ALK+, 51 (77%) were p-AKT+, and 55 (83%) were p-mTOR+. In the 5 p-p70S6K1 negative (p-p70S6K1-) cases, 2 (40%) were ALK+, 3 (60%) were p-AKT+, and 2 (40%) was p-mTOR+. The expression of p-p70S6K1 had no correlation to that of ALK and p-AKT (×2 = 0.119 and 0.684, respectively, p > 0.05), but was closely related to the expression of p-mTOR (χ2 = 4.295, p < 0.05; Table 3).

**Table 3:** Relationship between expression of p-4E-BP1 and p-p70S6K and activation of ALK, AKT, and mTOR

	**p-4E-BP1**		**p-value**	**p-p70S6K**		**p-value**
	**+ (n = 64)**	**- (n = 7)**		**+ (n = 66)**	**- (n = 5)**	
ALK (n = 71)						
ALK+	40	4		42	2	
ALK-	24	3	0.783	24	3	0.119
p-AKT (n = 71)						
p-AKT+	50	4		51	3	
p-AKT-	14	3	0.244	15	2	0.408
p-mTOR (n = 71)						
p-mTOR+	56	1		55	2	
p-mTOR-	8	6	0.000*	11	3	0.038*

*Statistically significant value.

### The interrelationship between the expression of ALK, p-AKT and p-mTOR and the clinical features

The median age of the 62 ALK + ALCL patients was 17 (4 ~ 68) years compared with 48 (16 ~ 74) years for the 41 ALK- cases. The age of onset was much younger in ALK + patients compared with ALK- cases (p < 0.01). In ALK + patients, 40 (65%) were male and 22 (35%) were female. In the 41 ALK- cases, 27 (66%) were male and 14 (34%) were female. Twenty-four (39%) of the ALK + patients had B symptoms compared with 14 (41%) of the ALK- cases. Fort-four (71%) ALK + patients had the primary lesion within the lymph node compared with 27 (66%) ALK- cases. Thirty-three (53%) ALK + patients were staging at III ~ IV compared with 20 (49%) ALK- cases. However, there were no statistical differences in sex distribution, clinical symptoms, primary lesion of the tumor, and Ann Arbor staging between ALK + and ALK- patients (p > 0.05).

The median age of the p-AKT + patients was 28 (4 ~ 74) years compared with 29 (4 ~ 65) years for the p-AKT- cases. In the 54 p-AKT + patients, 41 (76%) were male and 14 (24%) were female. In the 17 p-AKT- cases, 11 (82%) were male and 6 (18%) were female. Eighteen of the 54 (33%) p-AKT + patients had B symptoms compared with 6 of the 17 (36%) patients with p-AKT- tumors. Forty-two (78%) p-AKT + patients had the primary lesion within the lymph node compared with 12 (71%) p-AKT- cases. Twenty eight (52%) p-AKT + patients were staging at III ~ IV compared with 8 (47%) p-AKT- cases. There were no statistical differences in the age of onset, sex distribution, clinical symptoms, primary lesion of the tumor, and Ann Arbor staging between p-AKT + and p-AKT- patients (p > 0.05).

The median age of the 57 p-mTOR + patients was 25 (4 ~ 74) years compared with 40.5 (21 ~ 65) years of the 14 p-mTOR- cases. In the p-mTOR + patients, 44 (77%) were male and 13 (23%) were female, while in the 14 p-mTOR- cases, 11 (79%) were male and 6 (21%) were female. Twenty-two of the 57 (39%) p-mTOR + patients had B symptoms compared with 2 of the 14 (14%) patients with p-AKT- tumors. Forty-six (81%) p-mTOR + patients had the primary lesion within the lymph node compared with 8 (57%) p-mTOR- cases. Twenty-nine (51%) p-mTOR + patients were staging at III ~ IV compared with 7 (50%) p-mTOR- patients. Again, there was no statistical difference in the age of onset, sex distribution, clinical symptoms, primary lesion of the tumor, and Ann Arbor staging between p-mTOR + and p-mTOR- patients (p > 0.05).

On the whole, except that ALK + ALCL was predominantly seen at a younger age, there was no correlation between the expression of ALK, p-AKT, p-mTOR and its two downstream molecules, p-4E-BP1 and p-70S6K1, and the risk factors of the age of onset, sex distribution, clinical B symptoms, primary lesion of the tumor, and the Ann Arbor stage of the disease (Table 4).

**Table 4:** Relationship between the activation status of ALK, AKT and mTOR and the clinical features

	**ALK**		**p-value**	**p-AKT**		**p-value**	**p-mTOR**		**p-value**
	**+ (n = 62)**	**- (n = 41)**		**+ (n = 54)**	**- (n = 17)**		**+ (n = 57)**	**- (n = 14)**	
Age (years)									
Median	17	48		28	29		25	40.5	
Range	4–68	16–74	0.000*	4–74	4–65	0.877	4–74	21–65	0.076
Gender									
Male	40	27		41	14		44	11	
Female	22	14	0.889	13	3	0.573	13	3	0.912
Symptoms									
A	38	26		36	11		35	12	
B	24	15	0.828	18	6	0.889	22	2	0.068
Lesions									
Nodal	44	27		42	12		46	8	
Extranodal	18	14	0.583	12	5	0.547	11	6	0.07
Ann Arbor stage									
I ~ II	29	21		26	9		28	7	
III ~ IV	33	20	0.659	28	8	0.730	29	7	0.953

*Statistically significant value.

Symptom B, systemic symptoms of fever, night sweats, and weight loss; Symptom A, absence of the systemic symptoms.

### The prognostic significance of the expression of ALK, p-AKT, p-mTOR, p-4E-BP1, and p-p70S6K1

**Figure 2:**
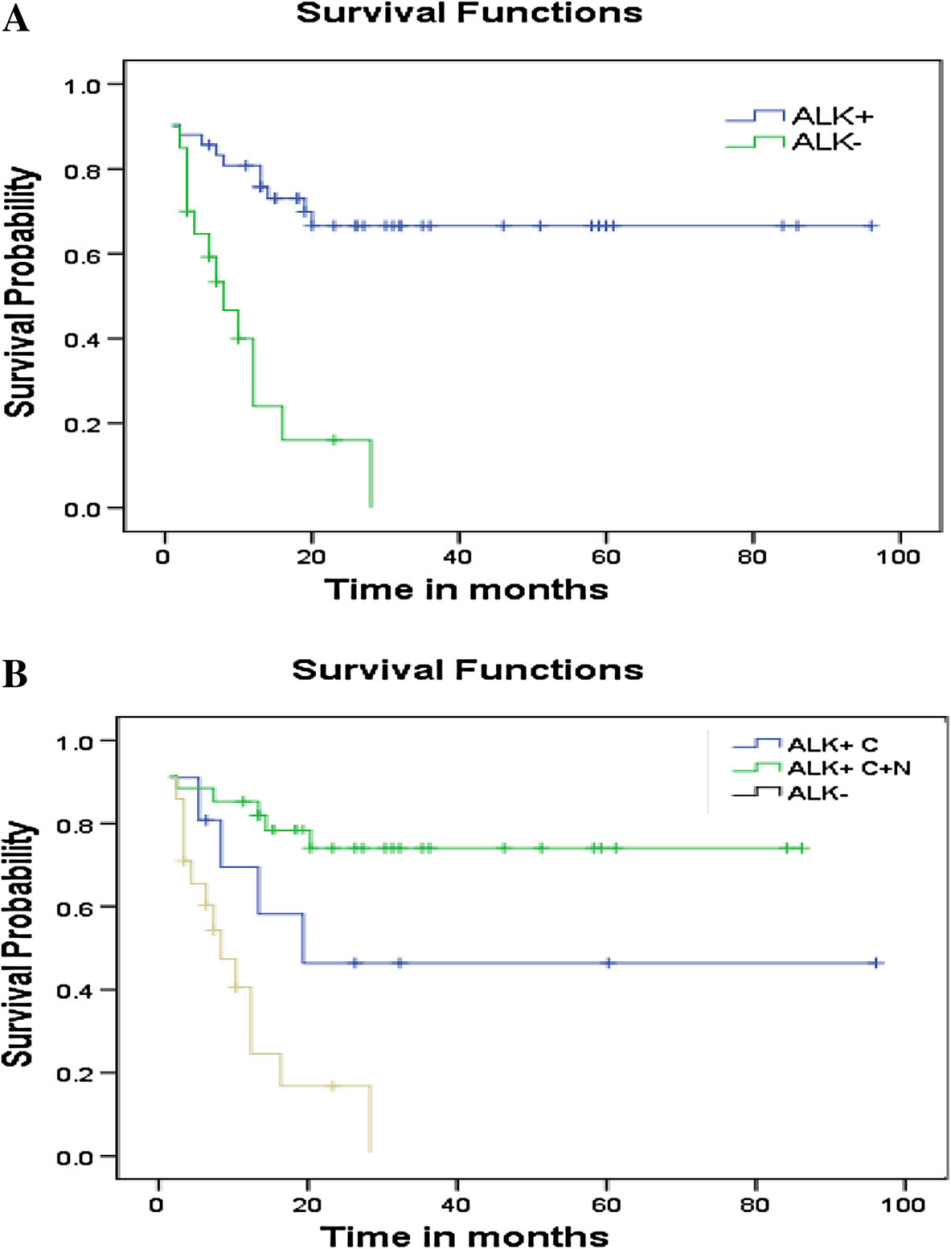
**Comparison of overall survival time in ALK + and ALK- ALCL patients. (A)** Kaplan-Meier curves showing significant difference in cumulative survival rate between ALK + and ALK- ALCL patients. **(B)** Kaplan-Meier curves showing no difference in cumulative survival rate between NPM-ALK + ALCLs and those ALCLs with variant ALK fusions.

Follow-up study was carried out in 70 of the 103 (68%) patients. The follow-up time was from 0.3 to 96.0 months. The survival time for ALK + patients ranged from 0.6 to 96.0 months and the median time was 27.0 months, with an expected 5-year survival rate of over 67%. The survival time for ALK- patients ranged from 0.3 ~ 28.0 months, with the median time of 4.0 months and a 2-year survival rate of 20%. The prognosis of ALK + patients was much better than that of ALK- cases. The difference was statistically significant (p < 0.01, Figure 2A), but there was no difference in the cumulative survival rate between NPM-ALK + ALCL patients and those with variant ALK fusions (Figure 2B). Compared with ALK expression, neither the expression of p-AKT, p-mTOR, p-4E-BP1, nor p-p70S6K1 had statistically significant differences regarding the median survival time and the 5-year survival rate in the ALCL patients.

Among other risk factors, including sex, age, primary lesion of the tumor (nodal or extranodal), Ann Arbor staging, histology, B symptoms, and immunological phenotype, only B symptoms had prognostic significance. By using Cox proportional hazards model analysis, only the expression of ALK and B symptoms had statistical significance on the prognosis (p < 0.05), of which the former had a greater impact than the latter.

### Overexpression of NPM-ALK activated the AKT/mTOR pathway in BaF3 cells and induced drug resistance to Dex and targeting AKT/mTOR re-sensitized the cell to Dex treatment

**Figure 3:**
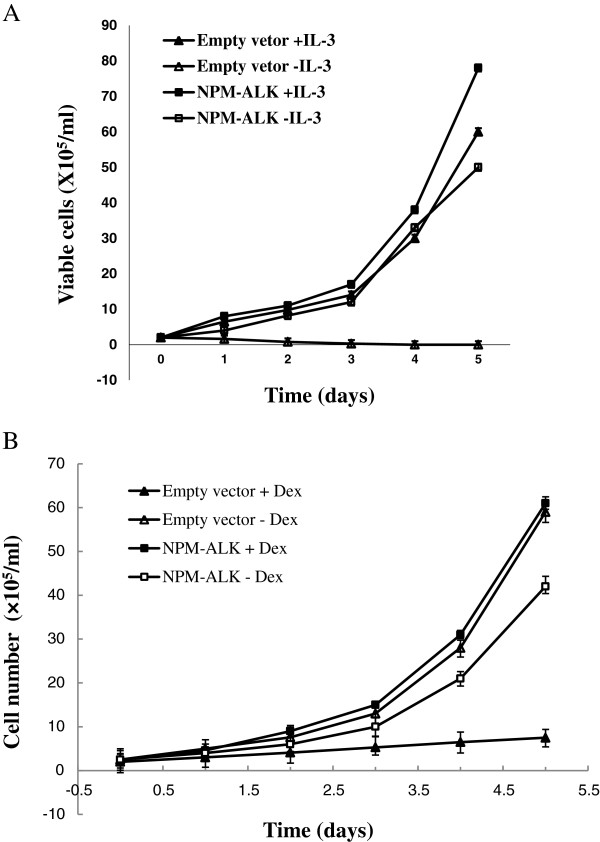
**Overexpression of NPM-ALK in BaF3 cells induced their cytokine independent growth and resistance to dexamethasone treatment. (A)** NPM-ALK confers IL-3-independent growth to BaF3 cells. Stably transfected BaF3 cells expressing NPM-ALK were assessed for growth in the presence or absence of IL-3, together with empty vector-containing cells. Viable cell counts were performed in triplicate using trypan blue at 24-hour intervals, with each point being the average of the triplicate measurements. **(B)** BaF3 cells transformed by NPM-ALK became resistant to GCs and could survive well in 1 μM Dex, while BaF3 cells transfected with empty vector could not proliferate at this concentration.

**Figure 4:**
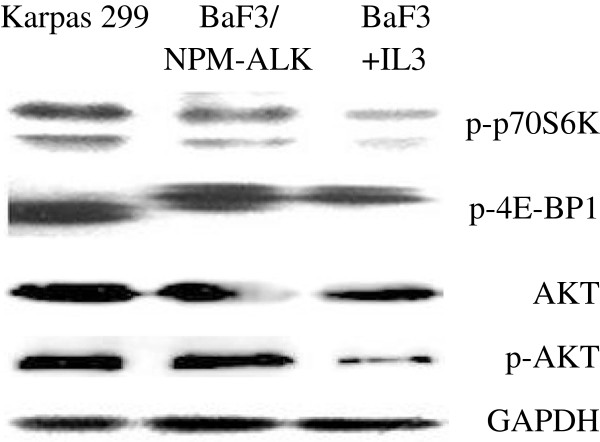
**Overexpression of NPM-ALK activated the AKT/mTOR pathway.** Overexpression of NPM-ALK in BaF3 cells induced hyper-activation of the AKT/mTOR pathway, as demonstrated by hyperphosphorylation of AKT and downstream molecules of mTOR: 4E-BP1 and p70SK6.

**Figure 5:**
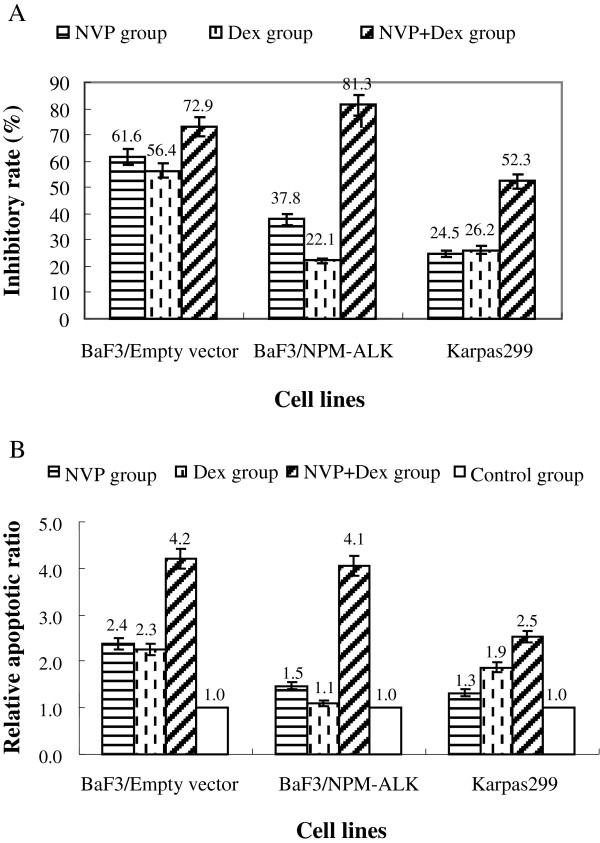
**Targeting AKT/mTOR had a therapeutic significance on ALK + tumor cells. (A)** Synergistic growth inhibition between the dual PI3K/mTOR inhibitor, NVP-BEZ235, and Dex in all cells, especially in BaF3/NPM-ALK. **(B)** A higher apoptosis rate in cells treated with NVP-BEZ235 and Dex together compared with single use of the drugs.

Expression of NPM-ALK converted BaF3 cells from IL-3 dependent to cytokine-independent growth, indicating a strong transforming activity of the kinase (Figure 3A). Interestingly, expression of NPM-ALK also conferred resistance to GC treatment. Compared with BaF3 cells transfected with empty vector, the transformed cells grew well at 1 μM Dex (Figure 3B) and could even survive at a concentration of Dex as high as 500 μM (data not shown). Western blotting analysis indicated the overexpression of NPM-ALK induced hyper-activation of the AKT/mTOR pathway in BaF3 cells, as demonstrated by hyperphosphorylation of AKT and downstream molecules of mTOR: 4E-BP1 and p70SK6 (Figure 4). Both Dex and the dual PI3K/mTOR inhibitor, NVP-BEZ235, had little effect on cell growth, but when they were used in combination a strong synergistic inhibitory effect was produced in ALK + cells, especially in the NPM-ALK transformed BaF3 cells (Figure 5A). Interestingly, when the kinase inhibitor was used in combination with Dex, it triggered apoptosis and convert the transformed BaF3/NPM-ALK cells re-sensitive to GC treatment (Figure 5B). The similar result was achieved when mTOR inhibitor rapamycin was used (data not shown).

## Discussion and conclusions

Anaplastic lymphoma kinase was first discovered by Dr. Morris and colleagues in 1994 due to its involvement in the t(2;5) chromosomal translocation in ALCL [24]. Since ALK expression is normally restricted to neural tissues, immunostainning of ALCL with ALK-specific antibodies becomes a quick, convenient, and also a reliable means to identify ALK + tumors. Large clinico-pathologic studies of ALCL have shown that about 80% of cases of ALK + ALCL exhibit cytoplasmic and nuclear staining indicative of the presence of NPM-ALK; whereas the remaining 20% express ALK proteins only in the cytoplasm of the tumor cells, indicating variant-ALK fusions [1,13,25]. Our study showed that 60% of ALCL tumors had ALK positive staining, of which 79% demonstrated a NPM-ALK staining pattern and 21% showed the staining pattern of variant-ALK fusions, which correlated well to the results in the literature [13,25,26]. We did not find any correlation between ALK expression and the histological subtype. In regard to the prognosis, there was no statistical difference between the patients with NPM-ALK and variant-ALK fusions. These data suggest that ALK + ALCLs have the same pathogenesis no matter what kind of ALK fusion partners they have.

The mean age of the 62 patients with ALK + ALCLs was 25 ± 15 years compared with 46 ± 17 years for the 41 ALK- cases. The former was much younger than the latter, p < 0.05. Although in our case there was a higher percentage of patients who had B symptoms and the primary lesion within the lymph nodes, or had the diseases at stage III ~ IV in ALK + than ALK- ALCLs, there was no statistical significance, p > 0.05. However, follow-up study indicated that ALK + ALCL patients had a much better prognosis than that of ALK- cases. The 5-year survival rate of ALK + cases was over 60%, whereas the 2-year survival rate for ALK- cases was only 20%. Kaplan-Meier curve and log-rank test showed that ALK + ALCL patients had a significantly better cumulative survival than ALK- ALCL cases (p < 0.05). This result is much corroborated by case studies elsewhere [13-16,26], except for the survival time. In our study the overall 5-year survival rate was 52% for all ALCL patients, 67% for ALK + cases, and less than 20% for ALK- patients compared with 77% for the total ALCLs, 80% for ALK + ALCLs, and 40% for ALK- ALCLs reported [7,8,25-27]. The cause for this difference is now under investigation.

Compiling evidence demonstrates that oncogenic NPM-ALK kinase induces the activation of mTOR signaling pathway, which contributes to NPM-ALK/PI3K/AKT-mediated tumorigenesis in ALCL, and that inhibition of AKT/mTOR represents a potential therapeutic strategy in ALK + ALCL [9,10,28]. In this study, activation of ALK/AKT/mTOR pathway was checked in 71 out of the 103 ALCL patient tumor samples. We found that the AKT/mTOR pathway was highly activated in ALK + ALCL. Phosphorylation of Thr308p-AKT and Ser2448p-mTOR was detected at higher percentages in ALK + ALCL tumors and their activation was closely related to ALK expression, but not related to its expression pattern, suggesting that no matter what the ALK fusions they are, they can activate the AKT/mTOR pathway. Our in vitro study also confirmed that NPM-ALK had strong transforming activity in lymphocytes and overexpression of NPM-ALK could induce the activation of the AKT/mTOR signaling pathway.

Activation of the AKT/mTOR pathway has been associated with aggressive disease and poor prognosis in certain cancers, like breast cancer [29-33]. However, currently there is little information on the prognostic value of the activation of the AKT/mTOR pathway in ALCL. We checked the phosphorylation status of AKT, mTOR, and its two downstream effectors, p70S6K1 and 4E-BP1, and studied its correlation with clinical risk factors. Compared with ALK expression, expression of p-AKT, p-mTOR, p-p70S6K1 and p-4E-BP1 had no correlation with clinical features such as age, sex, symptoms, primary lesions and tumor staging, or overall survival, indicating that activation of the AKT/mTOR pathway had no prognostic value in ALCL. However, our in vitro study indicated that inhibition of the AKT/mTOR pathway could effectively reverse the GC resistance induced by overexpression of NPM-ALK in lymphocytes. Considering GC is the most commonly used and highly effective drug used for decades in the treatment of lymphoid malignancies, targeting AKT/mTOR might be an attractive therapeutic goal in the future.

In summary, we have shown that the AKT/mTOR pathway was highly activated in ALK + ALCL. However, activation of this pathway does not confer any prognostic significance in ALCL as in some other tumors [34-37]. However, this does not compromise the therapeutic importance of blocking the AKT/mTOR pathway in this disease considering that activation of AKT/mTOR leads to resistance to chemo-reagents [38] and glucocorticoids [39] which constitute the first choice for the treatment of lymphoid malignancies including ALCL. Clinical use of AKT/mTOR inhibitors in the treatment of ALCL should be further explored.

## Abbreviations

ALCL: Anaplastic large cell lymphoma; ALK: Anaplastic lymphoma kinase; NPM: Nucleophosmin; mTOR: Mammalian target of rapamycin; MEK: Mitogen-induced extracellular kinase; ERK: Extracellular signal-regulated kinase; PI3K: Phosphatidylinositol 3-kinases; AKT: Protein kinase B; GC: Glucocorticoid; 4E-BP1: 4E-binding protein-1; p70S6K1: 70 kDa ribosomal protein S6 kinase polypeptide 1; TPM3: Tropomyosin 3; ATIC: 5-aminoimidazole-4-carboxamide ribonucleotide formyltransferase/IMP cyclohydrolase; IHC: Immunohistochemical; EMA: Epithelial membrane antigen; PBS: Phosphate-buffered saline; Dex: Dexamethasone; OS: Overall survival.

## Competing interests

The authors declare that they have no competing interests.

## Authors’ contributions

JG and MY contributed to the experimental design, specimen collection, data acquisition. YZ and LG participated in data analyses, interpretation of results. YZ, QL and CJ participated in the design of the study and carried out data interpretation. ZM contributed to conception, experimental design, data acquisition, analyses, and interpretation, and manuscript preparation. All authors read and approved the final manuscript.

## Pre-publication history

The pre-publication history for this paper can be accessed here:

http://www.biomedcentral.com/1471-2407/13/471/prepub
